# Genome-Wide Analysis of *bHLH* Family Genes and Identification of Members Associated with Cold/Drought-Induced Photoinhibition in *Kandelia obovata*

**DOI:** 10.3390/ijms242115942

**Published:** 2023-11-03

**Authors:** Junjian Li, Siyi Chen, Yaxin Yin, Qiaobo Shan, Chunfang Zheng, Yan Chen

**Affiliations:** 1 College of Life and Environmental Science, Wenzhou University, Wenzhou 325035, China; 2 College of Landscape Architecture and Forestry, Qingdao Agricultural University, Qingdao 266109, China; 3 Forestry College, Inner Mongolia Agricultural University, Hohhot 010018, China

**Keywords:** *Kandelia obovata*, basic Helix-Loop-Helix (*bHLH*), cold stress, drought stress, photoinhibition

## Abstract

Plant basic helix-loop-helix (*bHLH*) transcription factors play pivotal roles in responding to stress, including cold and drought. However, it remains unclear how *bHLH* family genes respond to these stresses in *Kandelia obovata*. In this study, we identified 75 *bHLH* members in *K. obovata*, classified into 11 subfamilies and unevenly distributed across its 18 chromosomes. Collineation analysis revealed that segmental duplication primarily drove the expansion of *KobHLH* genes. The *KobHLH* promoters were enriched with elements associated with light response. Through RNA-seq, we identified several cold/drought-associated *KobHLH* genes. This correlated with decreased net photosynthetic rates (*P*_n_) in the leaves of cold/drought-treated plants. Weighted gene co-expression network analysis (WGCNA) confirmed that 11 *KobHLH* genes were closely linked to photoinhibition in photosystem II (PS II). Among them, four Phytochrome Interacting Factors (PIFs) involved in chlorophyll metabolism were significantly down-regulated. Subcellular localization showed that *KobHLH52* and *KobHLH30* were located in the nucleus. Overall, we have comprehensively analyzed the *KobHLH* family and identified several members associated with photoinhibition under cold or drought stress, which may be helpfulfor further cold/drought-tolerance enhancement and molecular breeding through genetic engineering in *K. obovata*.

## 1. Introduction

Mangroves function as natural habitats for various organisms and provide ecosystem services such as biodiversity maintenance, coastal erosion prevention, and carbon sequestration [1,2]. Although mangroves play a critical role for the maintenance of the ecological environment, they are also threatened by extreme climate events, including cold and drought [3,4]. It is found that cold stress can lead to the wilting and even death of large numbers of mangrove plants at its latitudinal limit of distribution in China [5]. In 1972 to 2004, prolonged drought due to less rainfall increased the vulnerability of mangroves in response to changing climate conditions in Australia [6]. Recent researches revealed that drought stress restrained mangrove growth and development [7]. As a result, protecting and restoring mangrove forests under extreme climatic conditions remains a major challenge [8].

*K. obovata*, the most cold-tolerant mangrove in China, faces imminent threats from extreme climate events, notably cold and drought stresses [7]. For instance, drought stress results in suppressed water absorption, closed stomata, and hindered CO_2_ exchange in *K. abovata* [9,10]. Meanwhile, under cold stress, the expression levels of genes associated with photosynthesis, specifically photosystem I (PSI) and photosystem II (PSII), are significantly down-regulated in *K. obovata* [11]. Moreover, transcription factors (TFs) play a crucial role in regulating the expression of target genes during various biological processes [12]. However, it remains uncertain how TFs influence the physiological functions of *K. obovata* in response to cold and drought stress. The completion of *K. obovata* genome sequencing has opened avenues to explore these molecular mechanisms using genomic resources [13].

The bHLH TF family stands as the second-largest family of TFs in eukaryotes [12]. Each bHLH protein comprises two functionally distinct regions. The bHLH domain, situated at the N-terminus, is essential for DNA binding and recognizes CANNTG in the E-box [14]. The HLH region at the C-terminus consists of two alpha-helixes separated by a loop of variable length, allowing the formation of homodimers and heterodimers among bHLH proteins [15,16]. To date, *bHLH* family genes have been identified in various plants, including *Arabidopsis thaliana* [17], *Oryza sativa* [18], and *Malus domestica* [19]. Functional studies have shown the involvement of *bHLH*s in the regulation of growth, development, and stress responses [20,21]. Notably, *bHLH* genes are known to regulate photosynthesis [22]. For instance, Arabidopsis PIF1 negatively regulates chlorophyll biosynthesis by binding to a G-box motif (CACGTG) in the promoter of *Protochlorophyllide Oxidoreductase* (*PORC*) [23]. Additionally, poplar *PebHLH35* positively regulates stomatal density, stomatal aperture, and photosynthesis under drought stress [24]. However, the *bHLH* family in *K. obovata* has yet to be characterized.

*K. obovata* have a remarkable ability to sequester carbon, so many researchers are committed to move them northward to reduce the greenhouse effect. It is found that *K. obovata* was introduced to Yueqing Bay (28°20′ N), Zhejiang Province, where the propagules can grow into seedlings [25]. However, extreme drought and cold events exert a destructive influence on *K. obovata* [26]. Fortunately, the achievement of the *K. obovata* genome sequencing provides a new opportunity to explore the molecular mechanism using the genomic resources [13]. Thus, the goal of this study was to screen candidate *KobHLH* genes associated with the cold/drought-induced photoinhibition to promote molecular breeding through genetic engineering and increase the resistance to adversity.

In this study, we conducted a comprehensive analysis of *bHLH* genes in *K. obovata*, elucidating their gene structure and evolutionary relationships. Using transcriptomic data, we identified several *bHLH* genes responding to cold and drought stresses. Among these, several *KobHLH* genes were predicted to induce PSII photoinhibition in *K. obovata* under cold and drought stresses. Our study thereby contributes to the understanding of the role played by *KobHLH* genes in regulating the photosynthetic process under abiotic stress in *K. obovata*.

## 2. Results

### 2.1. Identification of KobHLH Genes in K. obovata

**Figure 1:**
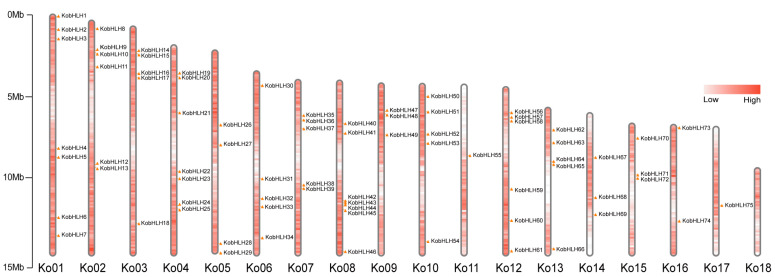
Chromosomal location of 75 *KobHLH* genes. Left scale represents the size of the chromosome, and the red areas represent the density of the genes on the chromosomes.

The *K. obovata* genome contained 75 *bHLH* genes, which were named *KobHLH1-KobHLH75* based on their positions on the chromosomes (Table S3). These *KobHLH* genes exhibited an uneven distribution across 17 out of the 18 chromosomes (Figure 1). Chromosomes 1, 4, and 8 contained the highest number (7, 9.3%) of *KobHLH* genes, whereas chromosome 17 had only one *bHLH* gene. No *KobHLH* genes were found on chromosome 18. Analyzing the properties of the 75 KobHLH proteins revealed variations in sequence structures and biochemical features (Table S3). Subcellular localization prediction indicated that 70 out of the 75 KobHLH proteins were located in the nucleus, whereas 2 were in the chloroplast, 1 in the mitochondrion, 1 in the vacuole, and 1 in the peroxisome.

### 2.2. Conserved Motifs and Gene Structure of KobHLH Proteins

**Figure 2:**
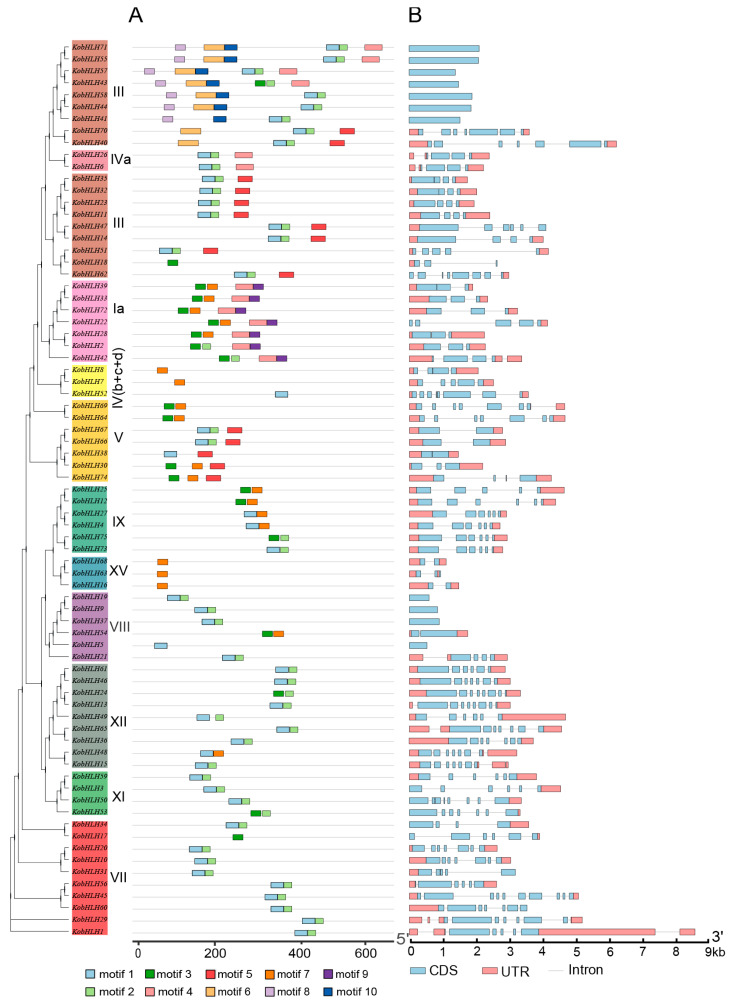
Conserved motifs and gene structures of the *KobHLHs.* (**A**) Ten motifs of KobHLH proteins are represented by different colored boxes. (**B**) Gene structure of *KobHLH* genes. The gray lines represent introns, red boxes represent the untranslated region (UTR), and blue boxes represent exons.

A total of 10 conserved motifs were identified in the 75 KobHLH proteins using the online MEME software (Table S4). These KobHLH proteins contained varying numbers of motifs. For instance, KobHLH71/55/57/43 had the largest number of motifs, whereas KobHLH18/8/7/52/5/17 had only one motif (Figure 2A). Most KobHLH proteins contained motif1, characterized by a basic region and a helix region. KobHLH proteins sharing identical motifs tended to cluster in the same subgroup, indicating potential functional similarities.

**Figure 3:**
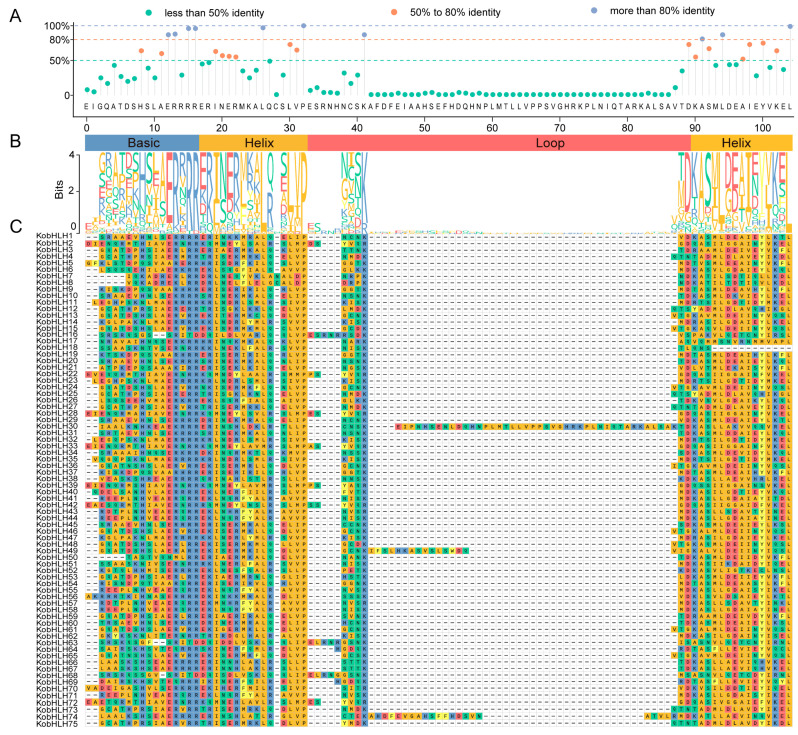
Schematic diagrams of the conserved amino acids and multiple alignment of the KobHLH domains. (**A**) The conserved amino acids of the KobHLH domains shown with the lollipop. (**B**) Sequence logo for the KobHLH domains. Height of each stack represents conservation of the sequence at a position. (**C**) Multiple sequence alignment of the KobHLH domains. Different colors represent different amino acids, red for D and E, green for N, C, Q, S and T, blue for R, H and K, orange for A, G, I, L, M, P and V, and gold for F, W and Y.

Sequence alignment of the bHLH domain in KobHLH proteins showed that 25 amino acids had consensus ratios exceeding 50%, and 10 amino acids exceeded 80% (Figure 3A). In the bHLH domain, Arg-16, Arg-17, Pro-33, and Leu-105 exhibited high sequence identity (>95%) (Figure 3C), implying potential roles in promoting dimerization.

*KobHLH* genes displayed various gene structures with different exon numbers (Figure 2B). Notably, 11 (14.7%) *KobHLH* genes were intron-less, 62 (82.7%) genes had more than two exons, and only 2 genes had more than 10 exons. Generally, intron–exon distribution patterns within a subfamily of *KobHLHs* were similar.

### 2.3. Phylogenetic Analysis of bHLH Genes in K. obovata and Arabidopsis

**Figure 4:**
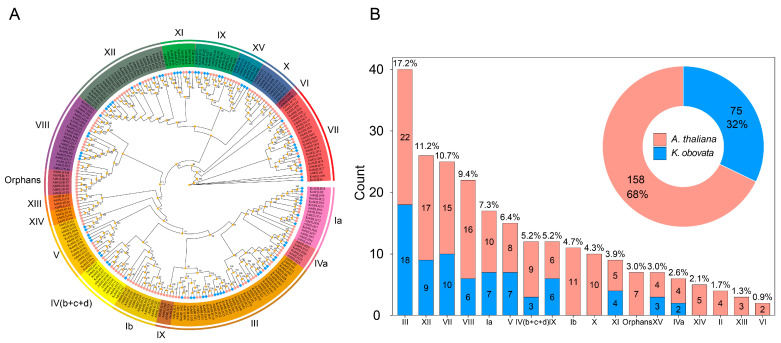
Phylogenetic analysis of KobHLH proteins from *K. obovata* and *A. thaliana*. (**A**) The unrooted phylogenetic tree of the KobHLH and AtbHLH proteins. Different subfamilies are marked with different colors. (**B**) Number of each subfamily in two species.

**Figure 5:**
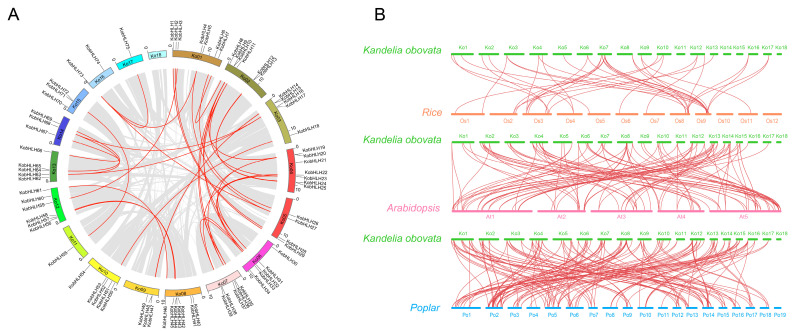
Syntenic analysis of bHLH genes from *K. obovata*, Arabidopsis, rice, poplar. (**A**) The collinearity of *K. obovata* genome shown by the gray-banded regions in the circle. The red line represents segmental duplications of *KobHLH* gene pairs. (**B**) Synteny relationships of the *KobHLH* genes with the homologous genes from three representative plants (Arabidopsis, rice, poplar).

The sequences of 75 KobHLH and 158 AtbHLH proteins were used to construct a phylogenetic tree. As shown in Figure 4A, KobHLH proteins were distributed in only 11 of the 18 AtbHLH subfamilies and were absent in subfamilies Ib, X, XIV, II, XII, and VI [27]. Subfamily III contained the most members (18), whereas subfamily IVa had the fewest (2) (Figure 4B). Out of the 75 *KobHLH* genes, 52 exhibited syntenic relationships. Specifically, 37 gene pairs were linked to segmental duplication, whereas no tandem duplication events were observed among paralogous genes (Figure 5A). The nonsynonymous (Ka) to synonymous (Ks) substitution rate(Ka/Ks) for the 37 gene pairs derived from segmental duplication was calculated to assess selective pressure. Among these pairs, 33 had Ka/Ks ratios <1, indicating purifying selection (Table S5). The divergence time for these gene pairs ranged from 8.93 to 101.5 Mya, with 22 (66.7%) gene duplication events occurring 10–20 Mya (Table S5).

To investigate the evolutionary relationships of *bHLH* genes in representative plants, 102, 38, and 162 *bHLH* genes were identified in Arabidopsis, rice, and poplar, respectively (Figure 5B). The results showed that the *bHLH* genes from *K. obovata* were evolutionarily closer to those from poplar. A total of 18 collinear *KobHLH* genes were found in the four species, indicating conserved roles during evolution (Table S6).

### 2.4. Cis-Element and GO Analysis of the KobHLHs

**Figure 6:**
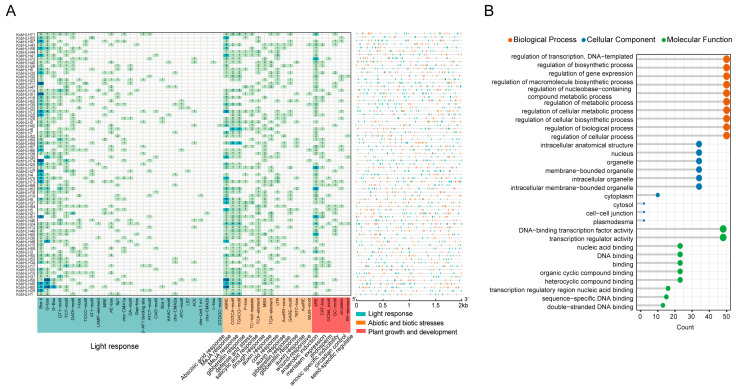
Identification of cis-acting elements and GO annotation of *KobHLH* genes. (**A**) Number of cis-acting elements in 2 kb promoter region of each *KobHLH* gene. The bluer color in the grids means more cis-acting elements, and the greener the fewer. The cis-acting elements in the *x*-axis are divided into three categories, with cyan representing light-responsive elements, orange representing stress-related elements, and red representing growth and development-related elements. The right side of the figure shows the position of the cis-acting elements in the promoter region. (**B**) Biological process, cellular component, and molecular function of *KobHLH* genes.

To identify the cis-acting elements present in the *KobHLH* promoters, 2 kb fragments upstream of the translation start sites for all 75 *KobHLHs* were extracted. These cis-elements were divided into three groups based on their functional roles. The most prevalent were light-responsive elements, including G-box and Box4, implying potential associations between some *KobHLH* genes and light responses (Figure 6A). Additionally, numerous cis-elements were linked to abscisic acid (ABA), gibberellin (GA), methyl jasmonate (MeJA), drought, cold, and wound responses. The third subgroup comprised elements like GCN4_moitf (associated with endosperm expression), CAT-box (related to meristem expression), and RY-element (involved in seed-specific regulation). Consistent with this, GO annotation revealed that the *KobHLH* genes were enriched in transcriptional regulation, gene expression, and metabolic biological processes (Figure 6B).

### 2.5. Expression Profiles of KobHLHs

**Figure 7:**
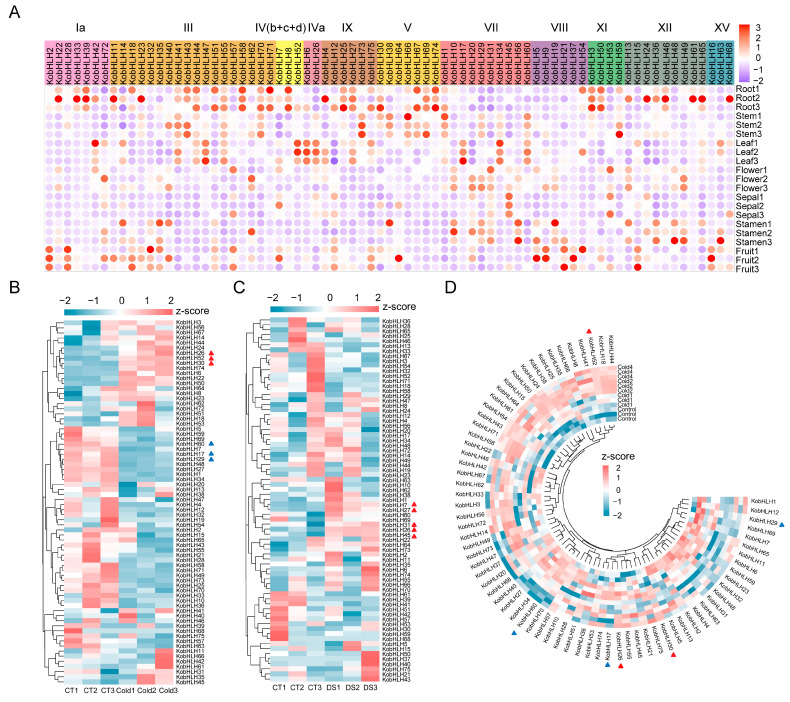
Expression patterns of the *KobHLH* genes. (**A**) Tissue expression patterns of the *KobHLH* genes. (**B**,**C**) The expression profiles of the *KobHLH* genes with cold or drought stress. (**D**) The expression profiles of the *KobHLH* genes in response to low temperature stress based on public transcriptomic data. The expression levels were normalized by row using a z-score method. The red and blue triangles indicate the genes that are significantly up-regulated and down-regulated, respectively, under cold and drought stress (*p* < 0.05).

Tissue expression patterns of the 75 *KobHLH* genes were analyzed using two transcriptomic data sets [11,28]. Generally, genes within the same subgroup exhibited similar expression patterns across various tissues (Figure 7A). For instance, subgroups III and V showed specific expression in roots and stems, whereas subgroup IVa showed specific expression in leaves. In contrast, most *KobHLH* genes exhibited negligible expression in flowers and stamens.

We further determined the responses of *KobHLH* genes to cold and drought stresses. As illustrated in Figure 7B, the expression levels of *KobHLH6*, *14*, *26*, *22*, *52*, *30*, *74*, *24*, *50*, and *53* were up-regulated, whereas those of *KobHLH29*, *59*, *69*, *60*, *7*, *17*, and *34* were down-regulated after cold treatment. These cold-responsive genes exhibited consistent expression patterns across three time points (Figure 7D). Similarly, the expression levels of *KobHLH7*, *27*, *31*, *26* and, *45* were induced, whereas those of *KobHLH52*, *18*, *71*, and *58* were repressed following drought treatment (Figure 7C). Six cold-responsive genes (*KobHLH52*, *30*, *26*, *29*, *17*, and *60*) and five drought-responsive genes (*KobHLH27*, *31*, *45*, *7*, and *26*) were validated via qRT-PCR (Figure S1), confirming the reliability of public RNA-Seq data.

### 2.6. KobHLH Genes Involved in Photosynthesis Metabolism

**Figure 8:**
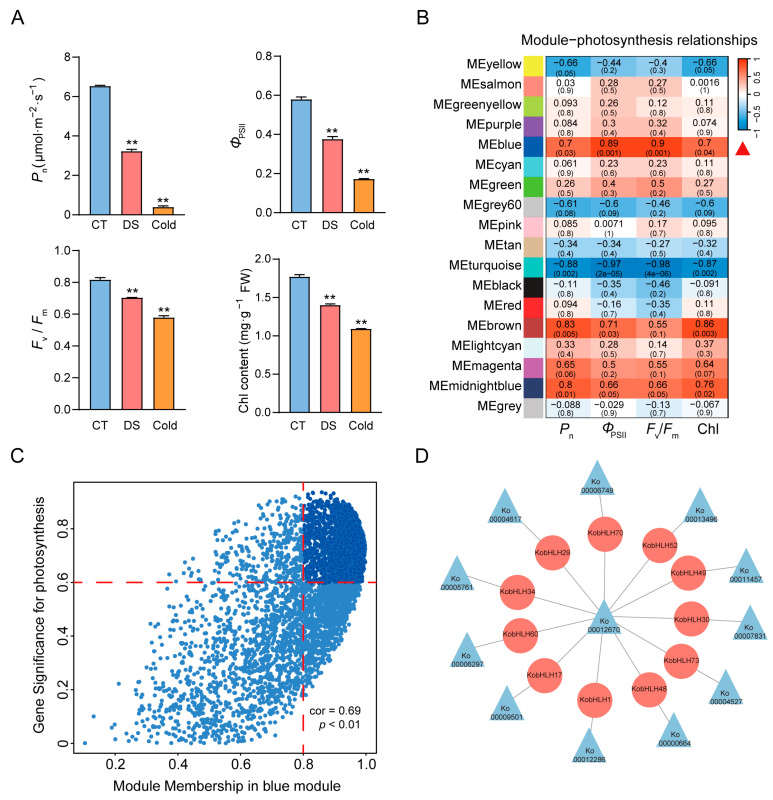
Gene modules and regulatory networks of *KobHLHs* associated with photosynthesis. (**A**) The net photosynthetic rate (*P*_n_), actual photochemical efficiency of PSII (*Φ*_PSII_), maximum photochemical efficiency of PSII (*F*_V_*/F*_m_), and total chlorophyll content (Chl) photosynthetic parameters in leaves of *K. obovata* seedlings under cold and drought conditions. The “**” represents statistically significant differences (*p* < 0.01) analyzed using Student’s *t*-test. (**B**) Heatmap of the correlation between the modules and photosynthesis. Each cell contains the corresponding Pearson correlation coefficient and p-value. Red represents a positive correlation, whereas blue represents a negative correlation. (**C**) Scatter plot of the blue module genes. The vertical and horizontal coordinate represent GS score and MM score for each gene. (**D**) The co-expression regulatory network of *KobHLH* genes associated with photosynthesis.

To determine whether cold and drought treatments affect the photosynthesis of *K. obovata* leaves, several photosynthetic parameters were detected. Net photosynthetic rate (*P*_n_)*,* stomatal conductance (*G*_s_)*,* actual photochemical efficiency of PSII (*Φ*_PSII_)*,* maximum photochemical efficiency of PSII (*F*_v_/*F*_m_), and chlorophyll content in the leaves significantly decreased, but minimum fluorescence (*F*_o_) increased after drought or cold treatment compared to the controls (Figure 8A; Figure S2). Moreover, cold stress had a more severe effect on leaf photosynthesis than drought stress.

To explore gene expression patterns associated with photosynthesis, a co-expression network was constructed using weighted gene co-expression network analysis (WGCNA). The expression profiles of 15,538 genes were divided into 18 modules, each containing 14 to 7304 genes (Figure 8B). The blue module exhibited a positive correlation with photosynthesis (*p* < 0.05). Within this module, 11 *KobHLH* genes were identified with |GS| > 0.6 and |MM| > 0.8 (Figure 8C), based on their co-expression relationships (Figure 8D). Cold and drought stress suppressed the expression of four *PIF* genes (*KobHLH1/17/29/60*) related to chlorophyll metabolism in *K. obovata*.

### 2.7. Subcellular Localization

**Figure 9:**
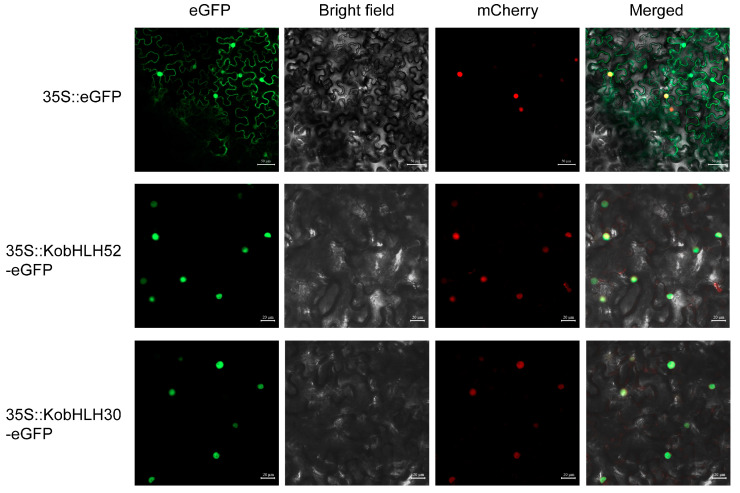
Subcellular localization of *KobHLH52* and *KobHLH30* in tobacco leaves.

Two photosynthesis-associated *bHLH* genes (*KobHLH52* and *KobHLH30*) from the blue module mentioned above were selected for subcellular localization analysis. Fluorescence signals in tobacco leaves transformed with 35S-*KobHLH52-eGFP* or 35S-*KobHLH30-eGFP* were observed in the nucleus (Figure 9), consistent with predictions using the WoLF PSORT software [29] (Table S2).

## 3. Discussion

bHLH gene family members have been extensively characterized in various species, including Arabidopsis [27], pepper [30], cucumber [31], poplar [32], and *Prunus mume* [15]. However, the study of *bHLH* gene in *K. obovata* has been limited. Advances in genome sequencing technologies have enabled a comprehensive analysis of the *bHLH* gene family in *K. obovata* [13]. In this study, 75 *bHLH* gene family members obtained from the *K. obovata* genome, and the number of *bHLH* genes in *K. obovata* was smaller than that in Arabidopsis (162) [17], rice (167) [18], and poplar (185) [32]. Although it has been reported that genome size might influence the number of *bHLH* genes in different species [33], *K. obovata* has a larger genome (180M) compared to *Arabidopsis* (125M). Hence, there might not be a correlation between the number of *bHLH* genes and genome size across plant species. Differences in genome assembly quality and screening criteria may account for the significant differences in the number of *bHLH* gene between *K. obovata* and poplar [15].

Gene duplication is a crucial mechanism governing the expansion and evolution of gene families in plants [34]. In this study, we found that 69% (52/75) of *KobHLH* genes, including 37 syntenic gene pairs, may have undergone segmental duplication, whereas no tandem duplication event was observed (Table S5), suggesting that the primary driving force behind the expansion of the *KobHLH* gene family is segmental duplication. Similar results have also been reported in other species, including *Raphanus sativus* and *Prunus mume* [15,22]. Furthermore, 89% of gene pairs stemming from segmental duplication exhibited Ka/Ks ratios less than 1 (Table S5), implying that these *KobHLHs* likely underwent strong purifying selection during their evolution [35,36].

Several *KobHLH* genes have been shown to participate in the regulation of organ size, flowering time, and stomatal opening [37,38]. In our study, we identified *KobHLH* genes with high expression in distinct tissues or stress responses. Notably, *KobHLH73*, *KobHLH45,* and *KobHLH2* displayed elevated expression in the stem, sepal, and fruit, hinting at their potential roles in these tissues (Figure 6). *KobHLH50* and *KobHLH70* exhibited high expressions in roots, and they are homologous to Arabidopsis *LRL1* and *GL3*, respectively, both of which positively regulate root development in Arabidopsis [39] (Figure 7). Additionally, *KobHLH4*, *KobHLH30,* and *KobHLH45* were responsive to abiotic stresses, such as cold, drought, and salt. In Arabidopsis, ICE1, a homolog of *KobHLH4*, improves cold tolerance by binding specifically to MYC sequences in the *CBF* promoter [40]. *SPT*, a homolog of *KobHLH30*, positively regulates the cold response [41]. *PIL5,* a homolog of *KobHLH45,* improves drought tolerance by increasing the contents of relative water content and chlorophyll levels [42].

After cold and drought stresses, photoinhibition can damage the photosynthetic electron transport system, resulting in reduced photosynthetic efficiency in plants [43,44]. In our study, we observed significant decreases in *P_n_*, *Φ_PSII_*, *F_v_/F_m_*, and Chl content in *K. obovata* leaves under cold and drought conditions, accompanied by an increase in *F_0_* (Figure 8, Figure S2). These results suggest that cold and drought treatments may cause PSII photoinhibition, resulting in a reduction in PSII efficiency in *K. obovata*. WGCN analysis revealed a blue module, including 11 *KobHLH* genes, which was associated with both photoinhibition and cold/drought responses (Figure 8). Among them, four *PIF* genes (*KobHLH1/17/29/60*) displayed reduced expression levels following cold or drought treatment, suggesting that cold-repressed *PIFs* may inhibit chlorophyll accumulation and chloroplast development, thereby affecting plant photosynthesis. Arabidopsis *bHLH41*, a homolog of *KobHLH52*, is known to regulate photosynthetic capacity by altering circadian rhythm [45]. Further experiments should be performed to explore the regulatory mechanisms of these *bHLH* genes in photosynthesis under cold or drought stress.

## 4. Materials and Methods

### 4.1. Identification of KobHLH Genes

The *K. obovata* genome sequence and annotation data were obtained from the Genome Sequence Archive database (https://ngdc.cncb.ac.cn/gsa/browse/CRA002395, accessed on 11 October 2022) [13]. The Hidden Markov Model (HMM) profile for the basic Helix-Loop-Helix (bHLH) domain (PF00010) was retrieved from the Pfam database (http://pfam.xfam.org/, accessed on 11 October 2022) [15]. Subsequently, the *bHLH* genes of *K. obovata* were searched in the protein database using HMM files (*e*-value ≤ 1 × 10^−5^). To obtain the bHLH protein sequences of *K. obovata,* bHLH protein sequences of *A. thaliana* were downloaded from the TAIR database (http://www.arabidopsis.org, accessed on 12 October 2022) and used to perform a BLASTP search with a cut-off *e*-value ≤ 1 × 10^−5^. After removing redundant genes, the conserved bHLH domain was searched using the National Center for Biotechnology Information (NCBI) Conserved Domain Search (https://www.ncbi.nlm.nih.gov/Structure/cdd/wrpsb.cgi, accessed on 13 October 2022), SMART (http://smart.embl-heidelberg.de, accessed on 13 October 2022), and Pfam. The properties of KobHLH proteins were predicted using ExPASy ProtParam (https://web.expasy.org/protparam/, accessed on 15 October 2022) [46]. Subcellular localization of KobHLHs was determined by the WoLF PSORT program (https://psort.hgc.jp/, accessed on 15 October 2022) [29]. The *KobHLH* genes were named according to their chromosomal location in the *K. obovata* genome.

### 4.2. Phylogenetic Analysis of KobHLHs

Multiple sequence alignments were performed using MAFFT 7.508 and then visualized using the R package ggmsa 1.8.0 [47,48]. The phylogenetic tree of *bHLH* genes from *K. obovata* and *A. thaliana* was constructed using the maximum-likelihood (ML) method generated by IQ-TREE 2.25 [49] with 1000 bootstrap replicates. *KobHLH* genes were classified into different subgroups following the classification of *AtbHLH* proteins [15,27].

### 4.3. Conserved Motif and Gene Structure of the KobHLHs

The identification of conserved motifs in *KobHLH* proteins was accomplished using the MEME Suite (http://meme.sdsc.edu/meme/itro.html, accessed on 20 October 2022). The maximum number (10) of motifs and an optimal width ranging from 6 to 200 residues were set [50]. Analysis of the exon/intron structures of the *KobHLH* genes was performed using the Gene Structure Display Server (GSDS, http://gsds.cbi.pku.edu.cn/, accessed on 20 October 2022) [51].

### 4.4. Chromosomal Distribution, Gene Duplication, and Syntenic Analysis of KobHLHs

The *KobHLH* genes were mapped onto chromosomes for visualization using the R package RIdeogram 1.78 [52]. Gene duplication of *KobHLH* genes was analyzed using the Multiple Collinearity Scan toolkit X version (MCScanX) with default parameters [53]. The Ka/Ks rates of homologous *KobHLH* genes were calculated using KaKs_Calculator 3.0 [36]. The Ks values were used to determine the dates of gene duplication events (T) based on the formula: T = Ks/2λ, where λ = 1.5 × 10^−8^ s [54]. Genome sequence and annotation information for Arabidopsis, rice, and poplar were downloaded from TAIR, RGAP (http://rice.uga.edu/, accessed on 10 October 2022), and Ensembl (http://plants.ensembl.org/index.html, accessed on 10 October 2022). Their syntenic relationships were analyzed and plotted using MCScanX.

### 4.5. Cis-Regulatory Elements in the KobHLH Promoters

The 2 kb promoter fragments of the *KobHLH* genes were extracted for analysis of cis-elements using PlantCARE (http://bioinformatics.psb.ugent.be/webtools/plantcare/html/, accessed on 10 November 2022) [55]. The KobHLH protein sequences were annotated using EGGNOG (http://eggnog-mapper.embl.de/, accessed on 10 November 2022) and GO (http://geneontology.org/, accessed on 10 November 2022). Enrichment analysis was conducted using the R package clusterProfiler 4.2.2 [56,57].

### 4.6. Plant Material and Treatments

The *K. obovata* seedlings were collected from the Quanzhou Bay Mangrove Nature Reserve (Fujian, China; 24°47′21″~24°59′50″ N). Propagules were potted in sand and watered with Hogland’s solution. The experiment followed a completely randomized block design, with three replicates for each treatment. Plants were grown at 20/25 °C (day/night), 75% humidity, and a 12/12 h (day/night) photoperiod, with a 400 μmol·m^−2^·s^−1^ photosynthetic photon flux density (PPFD).

One-year-old seedlings were exposed to drought stress at 60% or cold stress at −2/10 °C (day/night) for 4 days. The second pair of leaves was collected for RNA-Seq, qRT-PCR, and physiological assays. Seedlings at the eight-leaf stage were subjected to cold stress at 4 °C for 0, 1, 3, and 12 h in a climatic chamber at 20/25 °C (day/night), 75% humidity, and a 12/12 h (day/night) photoperiod, with 400 μmol·m^−2^·s^−1^ PPFD. The second pair of leaves was immediately collected and stored at −80 °C for qRT-PCR analysis. All treatments were performed with three biological replicates.

### 4.7. Measurement of Gas Exchange, Chlorophyll Fluorescence, and Chlorophyll Contents

The second fully expanded leaf was used for measurement of gas exchange parameters using a 400 μmol m^−2^ s^−1^ photosynthetic photon flux density (PPFD), net photosynthetic rate (*P*_n_), and stomatal conductance (*G*_s_) using a portable photosynthesis system (LI-6400, LI-COR Inc., Lincoln, NE, USA). Chlorophyll fluorescence parameters of *K. obovata* were measured with a pulse modulated fluorometer, recording initial fluorescence (*F*_o_) and the maximal fluorescence (*F*_m_). The maximal quantum efficiency of PSII (*F*_v_*/F*_m_) and actual efficiency of PSII (*Φ*_PSII_) were calculated as per the established methods [58]. Chlorophyll contents in the leaves were determined by spectrophotometry after 95% ethanol extraction [59].

### 4.8. RNA-Seq

Total RNA was extracted from one-year-old *K. obovata* leaves using the mirVana miRNA Isolation Kit (Ambion, Austin, TX, USA). Subsequently, libraries were prepared using the TruSeq Stranded mRNA LTSample Prep Kit (Illumina, San Diego, CA, USA) and sequenced on the Illumina sequencing platform (Illumina HiSeq X Ten). Paired-end reads were aligned to the *K. obovata* genome using HISAT2 (V. 2.1.0) [60]. The reads were quantified using featureCounts 2.0.6 [61], and gene expression levels were normalized using the transcripts per million (TPM) method.

Raw transcriptomic data from various *K. obovata* tissues (root, stem, leaf, and fruit) were obtained from the NCBI database (accession number: PRJNA416402). Additionally, RNA-Seq data for *K. obovata* leaves subjected to multiple episodes of cold stress were retrieved from the NCBI database (accession number: PRJNA678025) [11]. Expression levels of *KobHLHs* in transcripts per million mapped reads (TPMs) were collected for comprehensive transcriptomic data analysis [62].

### 4.9. Quantitative Real-Time PCR (qRT-PCR)

Total RNA was extracted from frozen leaves using Trizol reagent (Thermo Fisher Scientific, Waltham, MA, USA) following the manufacturer’s instructions. First-strand cDNA was obtained using a PrimeScriptTM 1st Strand cDNA Synthesis Kit. qRT-PCR was performed on a MA-6000 real-time PCR system using AceQ qPCR SYBR Green Master Mix (Vazyme Biotech, Nanjing, China). The primers are listed in Table S1. All experiments were performed in triplicate, and relative expression levels were calculated using the 2^−ΔΔCT^ method, normalized to 18S rRNA [63,64]. Statistical significance was assessed by one-way ANOVA tests with a significance level of *p*-value < 0.05.

### 4.10. Co-Expression Network Analysis (WGCNA)

Differentially expressed genes among the samples were selected for the weighted gene co-expression network analysis (WGCNA) [65]. The soft threshold β = 10 was chosen to construct the network based on the adjacency matrix. The adjacency matrix was transformed into a topological overlap matrix. Hierarchical clustering was used to identify modules containing at least 30 genes. The correlation between co-expression modules and photosynthetic traits was calculated to obtain vital modules. The absolute value of Pearson’s correlation between module membership (MM) and gene significance (GS) was used to select hub genes. Hub genes in vital modules were identified with |MM| > 0.8 and |GS| > 0.6 and visualized with Cytoscape 3.9.1 [66].

### 4.11. Subcellular Localization

Full-length cDNA of the *KobHLH* genes were amplified using the primer pairs (Table S2). Each fragment was inserted into the Nco Ⅰ site of pCAMBIA2301 and fused in-frame with enhanced green fluorescent protein (eGFP). The recombinant vector was transformed into *Agrobaeterium tumefaciena* strain GV3101 through heat shock. The abaxial surfaces of *Nicotiana benthamiana* leaves were injected with the suspension. Fluorescence was detected using a confocal laser-scanning microscope (Zeiss LSM980, Oberkochen, Germany).

## 5. Conclusions

This comprehensive study provides valuable insights into the *bHLH* gene family in *K. obovata.* A total of 75 *bHLH* genes were identified in *K. obovata* and classified into 11 subgroups. Within each subgroup, conserved motifs and gene structures displayed a general similarity. Our findings suggest that the expansion of the *KobHLH* gene family is likely attributed to segmental duplication events. Expression pattern analyses revealed six *KobHLH* genes associated with cold stress and five associated with drought stress. Notably, through WGCNA analysis, a regulatory network of 11 *KobHLH* genes was predicted to regulate photosynthesis metabolism. This study lays the groundwork for a deeper understanding of photosynthesis responses to cold and drought stress in *K. obovata.*

## Data Availability

Raw transcriptomic data had been deposited to the Genome Sequence Archive (GSA) in the National Genomics Data Center (NGDC) (CRA010510).

## Supplementary Materials

The following supporting information can be downloaded at: https://www.mdpi.com/article/10.3390/ijms242115942/s1.
