# Supplementary material for: Genome-Wide Analysis of bHLH Family Genes and Identification of Members Associated with Cold/Drought-Induced Photoinhibition in Kandelia obovata
Source: Int J Mol Sci. 2023 Nov 3;24(21):15942. doi: 10.3390/ijms242115942 (PMC10647802; doi:10.3390/ijms242115942)

Table S1. Primer pairs used for qRT-PCR analysis of *KobHLHs*

KobHLHs	Primer pairs	
	Forward primer (5'-3')	Reverse primer (5'-3')
KobHLH52	TTGATGACCATGTTCCGGGG	TCCTGGTAGAATTGCCGCTG
KobHLH30	GTTGGGCCGTATGAGTTCCA	CGCCTCTTCTCAGCTTCCTT
KobHLH26	GGAGCTTGGTGAACTGGGAA	CCTGCTGCATGTTCTCTCCA
KobHLH29	TCCGTCACTGTCACAACACC	ATTGAGTCCCCAGAAGCAGC
KobHLH17	CCGCAAACTCTCTCTCCGTT	GAAGGGCGGATGAACAAACG
KobHLH60	TCAATACCAAGCACCCGTCC	TTGTGAGAAGTTGGCACCGA
KobHLH31	TCTGTTGAGATTGACGCCGG	GCTCTATCAGATGGCACCCC
KobHLH45	GGTCCGGTGGTTATGCAGAA	GATGCTGTTGGTCTTGCTGC
KobHLH7	AAACCAGAATCCCACGGTCC	TGGTTGCCTGAAACATGGGA
KobHLH27	AAAGCAGAATCCAGGCACGA	TCCAGCAGGGGAGCTATTCT
KobHLH14	CGTCAAATCTCGGTGACCCA	TAAGGTGGGCTTTGGTTGCA
18S rRNA	GGGGCTCGAAGACGATCAGA	TTAAGCCGCAGGCTCCACTC

Table S2. Primer pairs used for subcellular localization analysis of *KobHLHs* in the *N. benthamiana* leaves

KobHLH	Primer pairs	
	Forward primer (5'-3')	Reverse primer (5'-3')
KobHLH52	ATGGACACCTTCTTTTCCATGG	CGTTGCCGACTTGGCAAAT
KobHLH26	ATGGCGATCTCATCCATCAGG	GTTCAACATTCAATTCGCTCATG

Table S3. Annotation of *bHLH* genes in *K. obovata*
(Please see separate Excel file)

Table S4. Top 10 Motifs of *KobHLHs*

Motif	E-value	Sites	Width	Multilevel consensus sequence
1	3.2×10^{-756}	50	36	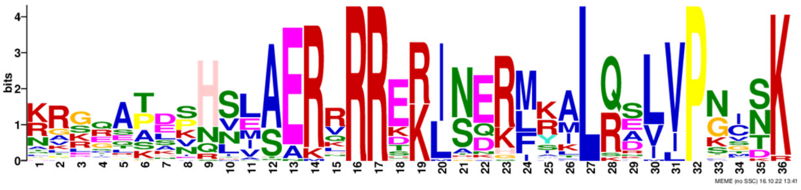
2	1.7×10^{-508}	50	23	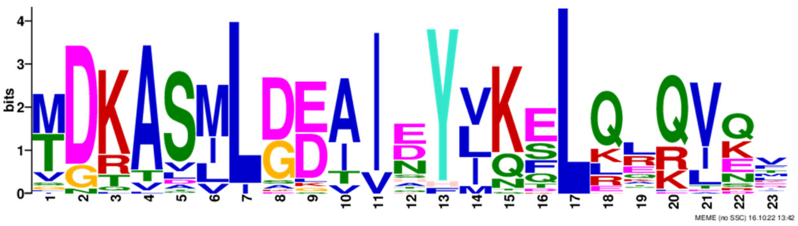
3	4.3×10^{-179}	20	29	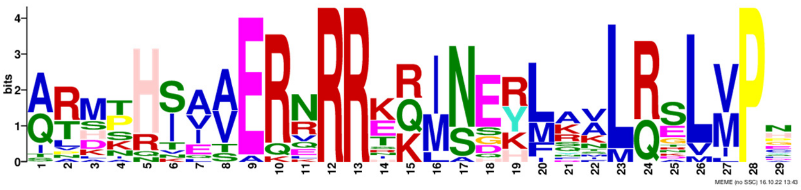
4	8.9×10^{-172}	13	49	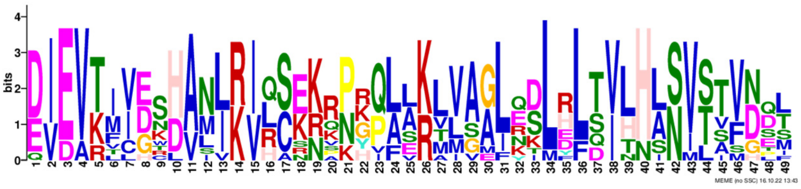
5	1.9×10^{-134}	15	41	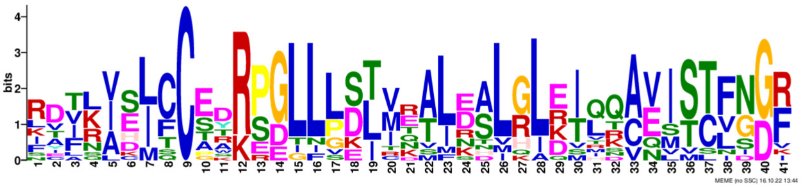
6	9.6×10^{-131}	8	57	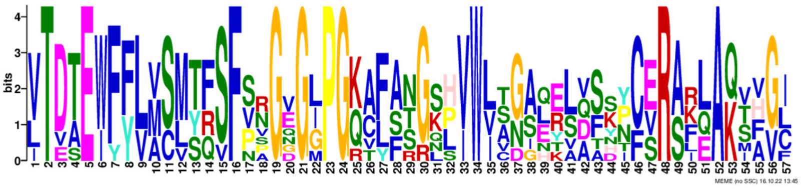
7	5.8×10^{-92}	20	29	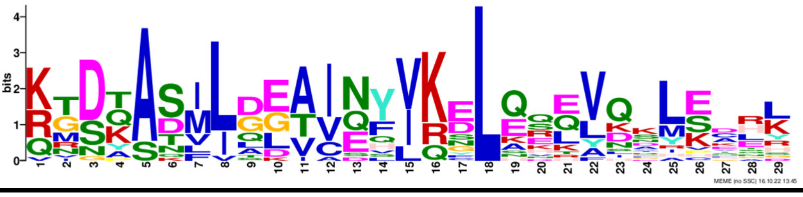

8	6.9×10^{-87}	7	29	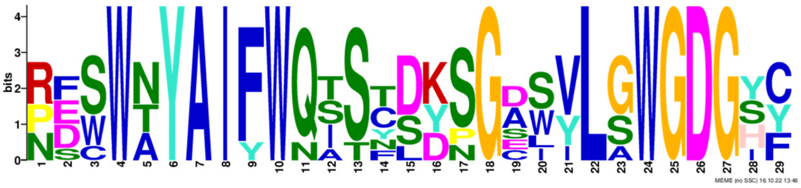
9	4.5×10^{-60}	7	29	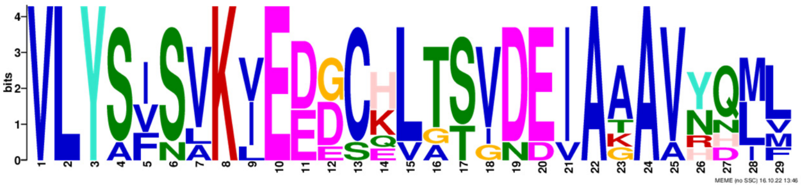
10	5.4×10^{-49}	7	36	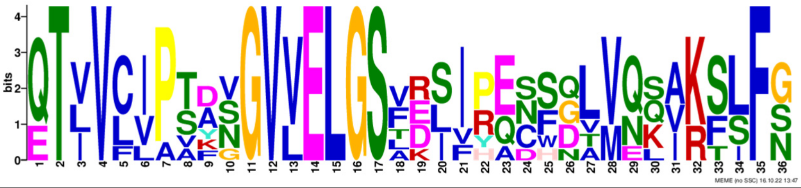

Table S5. The selection pressure and estimated divergence times for duplicate pairs of *KobHLHs*.

Duplication gene pair		Duplication	Ka	Ks	Ka/Ks	Divergence time (Million years ago)	Selection pressure
KobHLH27	*KobHLH73*	Segmental	0.5343	1.8835	0.2837	62.78	Purifying
KobHLH73	*KobHLH75*	Segmental	0.1316	0.4389	0.2997	14.63	Purifying
KobHLH19	*KobHLH37*	Segmental	-	-	-	-	-
KobHLH20	*KobHLH31*	Segmental	0.4732	2.3979	0.1973	79.93	Purifying
KobHLH23	*KobHLH32*	Segmental	0.2994	1.4437	0.2074	48.12	Purifying
KobHLH32	*KobHLH35*	Segmental	0.1128	0.3217	0.3507	10.72	Purifying
KobHLH63	*KobHLH68*	Segmental	0.0738	0.3885	0.1901	12.95	Purifying
KobHLH33	*KobHLH39*	Segmental	0.1123	0.3706	0.303	12.35	Purifying
KobHLH7	*KobHLH8*	Segmental	0.2987	2.3085	0.1294	76.95	Purifying
KobHLH6	*KobHLH26*	Segmental	0.1485	0.32	0.464	10.67	Purifying
KobHLH1	*KobHLH29*	Segmental	0.1637	0.2723	0.6014	9.08	Purifying
KobHLH2	*KobHLH28*	Segmental	0.1105	0.2788	0.3965	9.29	Purifying
KobHLH4	*KobHLH27*	Segmental	0.1161	0.268	0.4331	8.93	Purifying
KobHLH3	*KobHLH59*	Segmental	0.3689	1.7796	0.2073	59.32	Purifying
KobHLH4	*KobHLH73*	Segmental	0.4795	2.0907	0.2293	69.69	Purifying
KobHLH9	*KobHLH19*	Segmental	0.1257	0.5278	0.2381	17.59	Purifying
KobHLH10	*KobHLH20*	Segmental	0.1682	0.4136	0.4067	13.79	Purifying
KobHLH12	*KobHLH25*	Segmental	0.1082	0.5193	0.2084	17.31	Purifying
KobHLH13	*KobHLH24*	Segmental	0.1339	0.3191	0.4195	10.64	Purifying
KobHLH11	*KobHLH23*	Segmental	0.1092	0.3877	0.2816	12.92	Purifying
KobHLH10	*KobHLH31*	Segmental	0.4663	1.6259	0.2868	54.2	Purifying
KobHLH11	*KobHLH32*	Segmental	0.3522	1.9537	0.1803	65.12	Purifying
KobHLH9	*KobHLH37*	Segmental	-	-	-	-	-
KobHLH11	*KobHLH35*	Segmental	0.3476	1.5923	0.2183	53.08	Purifying
KobHLH14	*KobHLH47*	Segmental	0.0709	0.4647	0.1526	15.49	Purifying
KobHLH15	*KobHLH48*	Segmental	0.1369	0.29	0.472	9.67	Purifying
KobHLH18	*KobHLH51*	Segmental	0.1183	0.5081	0.2328	16.94	Purifying
KobHLH16	*KobHLH63*	Segmental	0.1564	1.5136	0.1033	50.45	Purifying
KobHLH16	*KobHLH68*	Segmental	-	-	-	-	-
KobHLH22	*KobHLH33*	Segmental	-	-	-	-	-
KobHLH22	*KobHLH39*	Segmental	0.4165	3.0451	0.1368	101.5	Purifying
KobHLH46	*KobHLH61*	Segmental	0.1413	0.4288	0.3295	14.29	Purifying
KobHLH43	*KobHLH57*	Segmental	0.1283	0.5205	0.2465	17.35	Purifying
KobHLH44	*KobHLH58*	Segmental	0.0996	0.4461	0.2233	14.87	Purifying
KobHLH55	*KobHLH71*	Segmental	0.0935	0.5508	0.1697	18.36	Purifying
KobHLH64	*KobHLH69*	Segmental	0.1685	0.4147	0.4062	13.82	Purifying
KobHLH66	*KobHLH67*	Segmental	0.0886	0.4142	0.2139	13.81	Purifying

Table S6. *KobHLHs* syntenic relationship with Arabidopsis, Rice, and Poplar

Kandelia obovata	Arabidopsis	Rice	Poplar
KobHLH1	*AT1G02340.1*	*LOC_Os05g04740.1*	*Po.05G000800*
KobHLH1	*AT1G09530.1*		*Po.13G002800*
KobHLH2			*Po.13G026300*
KobHLH3		*LOC_Os03g58330.1*	*Po.02G047700*
KobHLH3			*Po.05G058000*
KobHLH3			*Po.05G230500*
KobHLH3			*Po.13G043000*
KobHLH4	*AT2G42280.1*	*LOC_Os09g31300.1*	*Po.06G060000*
KobHLH4			*Po.16G054500*
KobHLH5	*AT5G01310.1*		*Po.06G111600*
KobHLH5			*Po.16G126400*
KobHLH6	*AT2G22750.2*		*Po.01G303700*
KobHLH6			*Po.07G011500*
KobHLH6			*Po.09G083700*
KobHLH7			*Po.04G171900*
KobHLH7			*Po.09G134600*
KobHLH8	*AT4G36060.1*		*Po.05G126500*
KobHLH8			*Po.09G134600*
KobHLH9	*AT3G50330.1*	*LOC_Os03g55550.1*	*Po.02G137800*
KobHLH9	*AT5G67060.1*		*Po.05G152400*
KobHLH9			*Po.07G053000*
KobHLH9			*Po.14G027200*
KobHLH10	*AT4G36930.1*		*Po.02G137100*
KobHLH10	*AT5G67110.1*		*Po.14G025900*
KobHLH11	*AT5G65640.1*		*Po.02G118700*
KobHLH11	*AT5G10570.1*		
KobHLH12	*AT1G05805.1*	*LOC_Os04g41229.1*	*Po.02G255100*
KobHLH12	*AT2G43140.2*		
KobHLH13			*Po.02G260000*
KobHLH13			*Po.14G157400*
KobHLH14		*LOC_Os01g70310.1*	*Po.12G113200*
KobHLH14			*Po.15G111800*
KobHLH15	*AT5G50915.1*	*LOC_Os08g42470.1*	*Po.01G068800*
KobHLH15		*LOC_Os09g33580.2*	*Po.03G176500*
KobHLH15			*Po.12G111900*
KobHLH15			*Po.15G110700*
KobHLH16	*AT3G47710.1*		*Po.12G072600*
KobHLH16	*AT5G39860.1*		*Po.15G066100*
KobHLH17	*AT5G61270.1*		
KobHLH16			*Po.17G084900*
KobHLH18			*Po.06G079900*
KobHLH18			*Po.18G146400*

KobHLH19	*AT3G50330.1*		*Po.02G137800*
KobHLH19	*AT5G67060.1*		*Po.05G152400*
KobHLH19			*Po.07G053000*
KobHLH19			*Po.14G027200*
KobHLH20	*AT4G36930.1*		*Po.02G137100*
KobHLH20	*AT5G67110.1*		*Po.14G025900*
KobHLH21	*AT1G66470.1*		*Po.04G096200*
KobHLH21	*AT5G37800.1*		*Po.17G136100*
KobHLH22	*AT5G65320.1*	*LOC_Os03g08930.1*	*Po.02G111600*
KobHLH22	*AT5G65640.1*	*LOC_Os09g29360.1*	*Po.05G077100*
KobHLH22			*Po.07G104100*
KobHLH23	*AT5G10570.1*		*Po.02G118700*
KobHLH24	*AT3G23690.1*		*Po.02G260000*
KobHLH24			*Po.14G157400*
KobHLH25	*AT2G43140.2*	*LOC_Os04g41229.1*	*Po.02G255100*
KobHLH25			*Po.14G159100*
KobHLH26	*AT2G22750.2*		*Po.01G303700*
KobHLH26			*Po.09G083700*
KobHLH27	*AT2G42280.1*	*LOC_Os09g31300.1*	*Po.06G060000*
KobHLH27			*Po.09G066400*
KobHLH27			*Po.16G054500*
KobHLH28			*Po.05G043100*
KobHLH28			*Po.13G026300*
KobHLH29	*AT1G09530.1*		*Po.05G000800*
KobHLH29	*AT1G02340.1*		*Po.13G002800*
KobHLH30	*AT2G41130.1*		*Po.06G037200*
KobHLH30	*AT3G56770.1*		*Po.16G038200*
KobHLH30	*AT3G56770.1*		
KobHLH31	*AT5G67110.1*		*Po.02G137100*
KobHLH31			*Po.05G153000*
KobHLH31			*Po.14G025900*
KobHLH32	*AT5G65640.1*		*Po.02G118700*
KobHLH33	*AT1G22490.1*	*LOC_Os03g08930.1*	*Po.02G111600*
KobHLH33	*AT5G65320.1*	*LOC_Os09g29360.1*	*Po.05G077100*
KobHLH34			*Po.02G157000*
KobHLH35	*AT5G65640.1*		*Po.02G118700*
KobHLH36	*AT1G10120.1*	*LOC_Os03g12940.1*	*Po.02G125300*
		LOC_Os03g51910.1	
KobHLH37	*AT3G50330.1*	*LOC_Os08g36740.1*	*Po.02G137800*
KobHLH37	*AT5G67060.1*	*LOC_Os09g28210.1*	*Po.05G152400*
KobHLH37			*Po.07G053000*
KobHLH37			*Po.14G027200*
KobHLH38		*LOC_Os08g37290.1*	*Po.02G115300*
KobHLH39	*AT1G22490.1*	*LOC_Os09g28900.1*	*Po.02G111600*

KobHLH39		*LOC_Os02g52190.1*	*Po.07G104100*
		LOC_Os03g08930.1	
		LOC_Os09g29360.1	
KobHLH40			*Po.02G059600*
KobHLH40			*Po.05G220300*
KobHLH41	*AT2G46810.1*		*Po.02G044400*
KobHLH41	*AT3G61950.1*		*Po.05G233500*
KobHLH42	*AT4G01460.1*		*Po.02G199200*
KobHLH42	*AT4G00870.1*		*Po.14G109600*
KobHLH42			
KobHLH43			*Po.01G086900*
KobHLH43			*Po.02G196200*
KobHLH43	*AT1G01260.1*		*Po.03G159800*
KobHLH43	*AT2G46510.1*		*Po.14G107000*
KobHLH44			*Po.02G196200*
KobHLH44	*AT2G20180.2*		*Po.02G191900*
KobHLH44	*AT4G28790.1*		*Po.14G101600*
KobHLH45	*AT3G07340.1*		*Po.02G279700*
KobHLH45	*AT5G48560.1*		
KobHLH46		*LOC_Os08g41320.1*	*Po.02G275500*
KobHLH46		*LOC_Os09g32510.4*	
KobHLH47	*AT5G50915.1*	*LOC_Os11g32100.1*	*Po.12G113200*
KobHLH47			*Po.15G111800*
KobHLH48		*LOC_Os08g42470.1*	*Po.01G068800*
KobHLH48			*Po.03G176500*
KobHLH48	*AT5G62610.1*		*Po.12G111900*
KobHLH48			*Po.15G110700*
KobHLH49			*Po.10G032900*
KobHLH49	*AT2G24260.1*		*Po.12G076300*
KobHLH49	*AT4G30980.1*		*Po.15G071000*
KobHLH50	*AT5G58010.1*	*LOC_Os02g55250.1*	*Po.06G195600*
KobHLH50	*AT4G29930.3*		*Po.18G114100*
KobHLH50			
KobHLH51	*AT5G56960.1*		*Po.06G079900*
KobHLH51			*Po.18G146400*
KobHLH52		*LOC_Os01g09900.1*	*Po.06G162000*
KobHLH53	*AT1G32640.1*	*LOC_Os06g09370.1*	*Po.06G149100*
KobHLH54	*AT4G17880.1*		*Po.01G202700*
KobHLH55	*AT5G46760.1*		*Po.01G153100*
KobHLH55	*AT5G46830.1*		*Po.03G098000*
KobHLH55	*AT2G46970.1*		
KobHLH55	*AT4G00870.1*		
KobHLH56			*Po.14G115200*
KobHLH57			*Po.01G086900*

KobHLH57	*AT1G01260.1*		*Po.02G196200*
KobHLH57	*AT2G46510.1*		*Po.14G107000*
KobHLH58	*AT1G03040.1*		*Po.02G191900*
KobHLH58	*AT4G02590.1*		*Po.14G101600*
KobHLH59		*LOC_Os03g58330.1*	*Po.02G047700*
KobHLH59		*LOC_Os07g08440.1*	*Po.02G052300*
KobHLH59			*Po.05G230500*
KobHLH59			*Po.05G058000*
KobHLH59			*Po.13G043000*
KobHLH60	*AT3G07340.1*		*Po.02G060800*
KobHLH60	*AT5G48560.1*		*Po.05G218700*
KobHLH61	*AT1G10610.1*	*LOC_Os08g41320.1*	*Po.02G275500*
KobHLH61	*AT1G12860.1*		
KobHLH62	*AT1G26945.1*		*Po.08G219500*
KobHLH62	*AT3G28857.1*		
KobHLH63	*AT3G47710.1*	*LOC_Os02g51320.1*	*Po.12G072600*
KobHLH63	*AT5G15160.1*		*Po.17G084900*
KobHLH63	*AT1G69010.1*		
KobHLH63			
KobHLH64			*Po.08G132600*
KobHLH64	*AT1G68920.1*		*Po.10G139000*
KobHLH64	*AT1G26260.1*		*Po.17G104300*
KobHLH65	*AT1G68810.1*		*Po.08G134000*
KobHLH65	*AT3G25710.1*		*Po.10G137500*
KobHLH66	*AT1G68810.1*		*Po.08G136900*
KobHLH66	*AT3G25710.1*		*Po.10G130000*
KobHLH67	*AT1G26945.1*		*Po.08G136900*
KobHLH67	*AT3G28857.1*		*Po.10G130000*
KobHLH68	*AT5G15160.1*		*Po.12G072600*
KobHLH68	*AT5G39860.1*		*Po.17G084900*
KobHLH68	*AT1G69010.1*		
KobHLH68	*AT5G08130.5*		
KobHLH69	*AT1G63650.1*		*Po.10G139000*
KobHLH69	*AT5G41315.1*		
KobHLH70			*Po.01G109500*
KobHLH70			*Po.02G176100*
KobHLH70	*AT1G32640.1*		*Po.03G137000*
KobHLH70	*AT4G17880.1*		*Po.14G085600*
KobHLH71	*AT5G46830.1*		*Po.01G153100*
KobHLH71	*AT5G46760.1*		*Po.03G098000*
KobHLH71	*AT5G46690.1*		
KobHLH71			
KobHLH72	*AT1G51140.1*		*Po.01G151900*
KobHLH72			*Po.03G099000*

KobHLH73		*LOC_Os08g39630.1*	*Po.01G018700*
KobHLH73		*LOC_Os09g31300.1*	*Po.01G287100*
KobHLH73			*Po.03G225600*
KobHLH73			*Po.06G060000*
KobHLH73			*Po.09G066400*
KobHLH73			*Po.16G054500*
KobHLH74	*AT1G51140.1*		*Po.04G060100*
KobHLH74			*Po.11G061200*
KobHLH75		*LOC_Os08g39630.1*	*Po.01G018700*
KobHLH75		*LOC_Os09g31300.1*	*Po.03G225600*

Figure S1. qRT-PCR analysis of 6 low temperature and 5 drought response *KobHLHs*. The "*" and "**" represent statistically significant differences ($p < 0.05$ and $p < 0.01$) analyzed using Student's t-test.

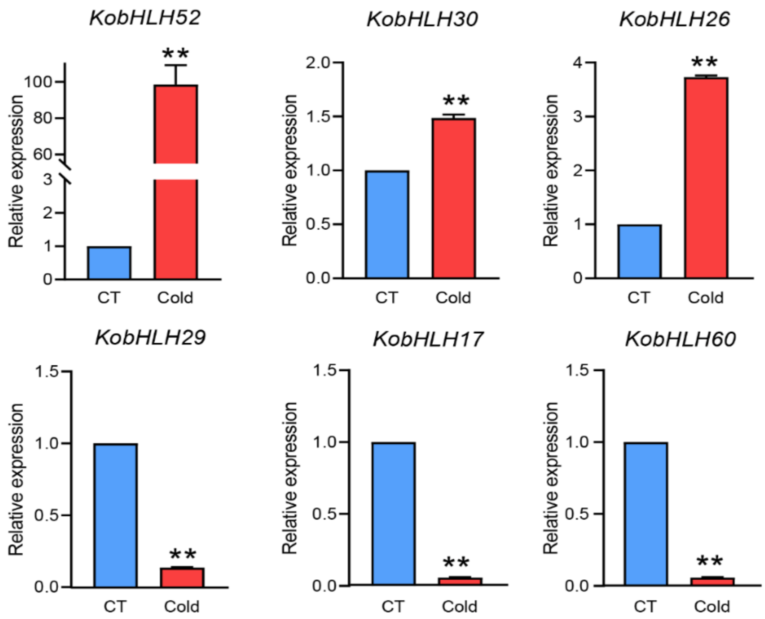

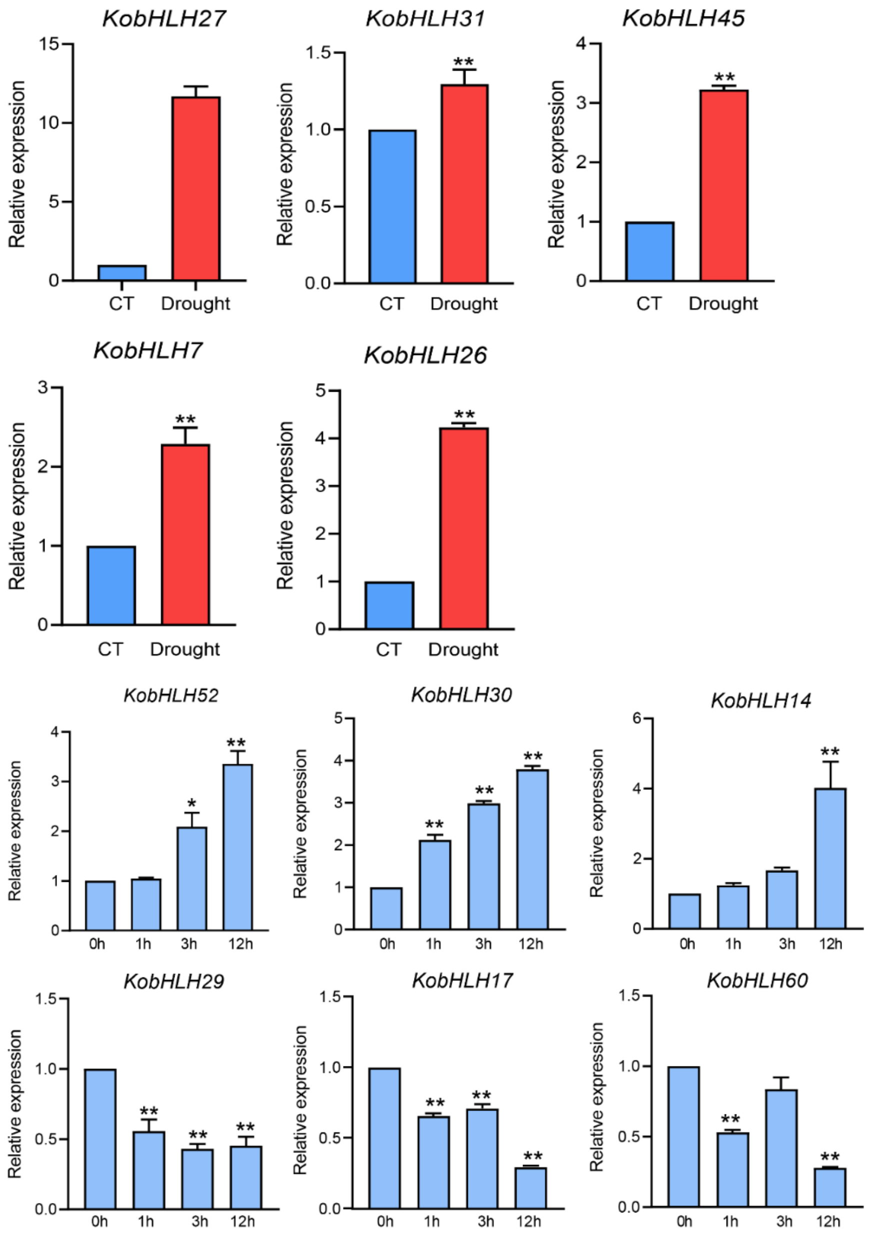

Figure S2. (A,B) gas exchange and minimum fluorescence parameters under drought and cold stress. The "**" represents statistically significant differences ($p < 0.01$) analyzed using Student's t-test.

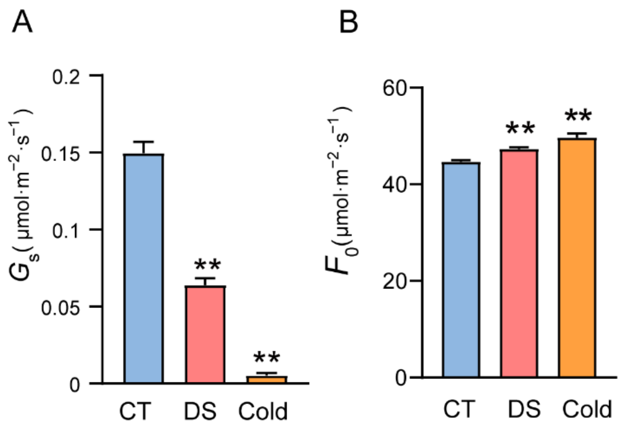

Supplement: Supplementary file 1 [file ijms-24-15942-s001.zip › Supplementary file.pdf]
